# AbAgKer: a unified semi-supervised framework for antigen-antibody binding affinity and kinetics prediction

**DOI:** 10.1093/bioinformatics/btag606

**Published:** 2026-08-12

**Authors:** Gang Luo, Junkai Wang, Sizhe Zhang, Zhilin Zhu, Zhangli Lu, Min Li

**Affiliations:** School of Computer Science and Engineering, Central South University, Changsha 410083, China; School of Computer Science and Engineering, Central South University, Changsha 410083, China; School of Computer Science and Engineering, Central South University, Changsha 410083, China; School of Computer Science and Engineering, Central South University, Changsha 410083, China; School of Computer Science and Engineering, Central South University, Changsha 410083, China; School of Computer Science and Engineering, Central South University, Changsha 410083, China

## Abstract

**Motivation:**

The rapid advancement of generative artificial intelligence has enabled the high-throughput design of therapeutic antibody candidates. However, the precise evaluation of these candidates remains a significant challenge due to the scarcity of high-quality activity data and the structural flexibility of antibody complementarity-determining regions (CDRs).

**Results:**

To address these challenges, we propose AbAgKer, an antibody screening model leveraging pre-trained representations and biological prior guidance for antigen-antibody affinity and kinetics prediction. Specifically, we design a biological prior-guided feature fusion framework that integrates pseudo-structural epitope knowledge and CDR-specific attention mechanisms via a mixture-of-experts architecture to effectively capture complex binding landscapes. To mitigate data scarcity, we employ a semi-supervised learning strategy for data self-distillation, which significantly enhances affinity prediction performance. Additionally, we demonstrate that the interaction representations learned by AbAgKer can be effectively transferred to the data-scarce task of predicting dissociation rates via few-shot learning. Extensive experiments demonstrate that AbAgKer outperforms baseline models and exhibits strong generalization capabilities in antibody screening and drug residence time analysis.

**Availability and implementation:**

The source code and dataset are available at https://github.com/CSUBioGroup/AbAgKer and https://doi.org/10.5281/zenodo.19691211.

## 1 Introduction

The development of therapeutic antibodies has revolutionized modern medicine, offering precise interventions for diseases ranging from cancer to viral infections (Pantaleo *et al*. 2022, Paul *et al*. 2024). With the advent of deep learning, antibody discovery is shifting from labor-intensive experimental screening to antibody de novo design. Recent years have witnessed an explosion of antibody generative models, ranging from sequence-based language models (Shuai *et al*. 2021, He *et al*. 2024, Wasdin *et al*. 2025) to structure-based diffusion models (Luo *et al*. 2022, Wang *et al*. 2024, Team *et al*. 2025), capable of generating millions of antibody candidates. The pre-trained antibody generative large language model (PALM-H3) (He *et al*. 2024) focuses specifically on sequence design for the heavy chain complementarity-determining region 3 involved in antigen-antibody interactions, successfully generating antibodies targeting SARS-CoV-2. Furthermore, DiffAb (Luo *et al*. 2022) emerged as the first deep learning model to generate antibodies targeting specific antigen structures. RFantibody (Bennett *et al*. 2026) fine-tunes RFdiffusion (Watson *et al*. 2023) on antibody complex structure data to achieve precise sampling of complementarity-determining regions (CDRs) loops targeting specified epitopes, with binding states confirmed via cryo-electron microscopy. However, the lack of accurate, high-throughput computational screening tools to evaluate these generated candidates remains a significant challenge (Cheng *et al*. 2024).

**Figure 1:**
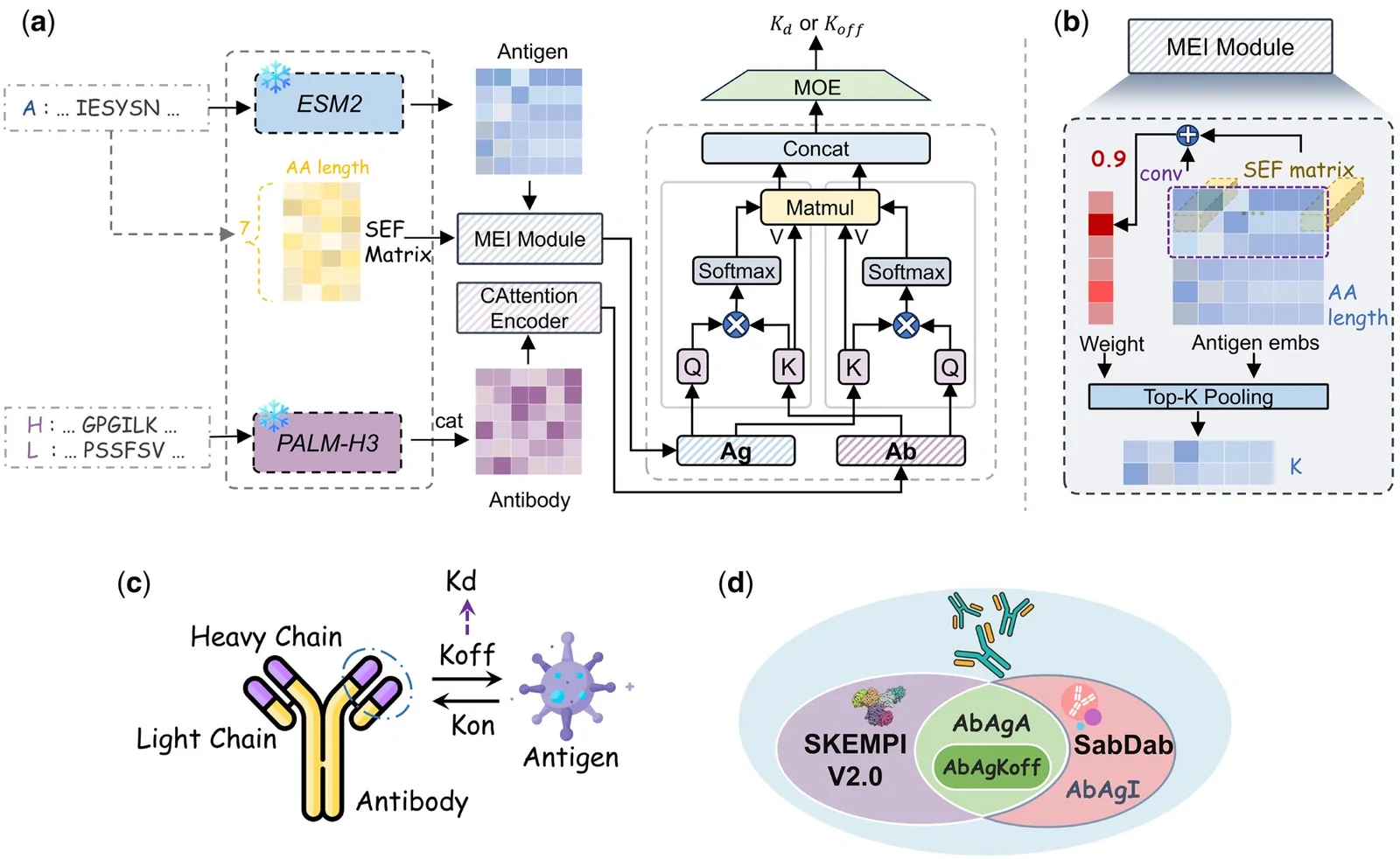
(a) The overall framework of AbAgKer. (b) The pipeline of the MEI Module. (c) Antigen-antibody binding affinity is typically quantified by the equilibrium dissociation constant ($K_{d}$), which is derived from the kinetic association ($k_{on}$) and dissociation ($k_{\mathrm{off}}$) rate constants. (d) Based on the SKEMPI v2.0 and SAbDab databases, we constructed three datasets for this study: the affinity dataset AbAgA, the interaction dataset AbAgI, and the antibody dissociation rate dataset AbAgKoff.

The gold standard for in silico verification is binding affinity prediction, which predicts the equilibrium dissociation constant ($K_{d}$) to reflect the strength of the interaction. With the advent of Protein Language Models (PLM), recent affinity prediction studies have attempted to model affinity based on domain-specific pre-trained representations, such as AntiBERTy (Ruffolo *et al*. 2021) and AntiBERTa2 (Barton *et al*. 2024). For instance, DG-Affinity (Yuan *et al*. 2023) employs ConvNeXt to integrate representations from the general protein model TAPE (Rao *et al*. 2019) and the antibody-specific model AbLang (Olsen *et al*. 2022). Similarly, prior study (He *et al*. 2024) proposed a high-precision predictor by fusing its pre-trained antibody features with antigen ESM-2 (Evolutionary Scale Modeling 2) representations to evaluate binding strength against SARS-CoV-2. Meanwhile, MVSF-AB (Li *et al*. 2025) utilizes residue AAindex features and ProteinBERT (Brandes *et al*. 2022) antibody representations, and achieves excellent correlation performance across multiple datasets. However, the scarcity of high-quality data continues to limit the development of this field. Moreover, antigen-antibody affinity prediction is insufficient to screen for excellent antibodies in the real world. The dissociation rate ($k_{\mathrm{off}}$) provides critical kinetic information (Vauquelin 2016) in antigen-antibody binding, as shown in Fig. 1c, revealing the stability of the complex and the duration of biological action. Even for interactions with identical $K_{d}$ values, varying $k_{\mathrm{off}}$ can lead to significant differences in biological behaviors, such as drug efficacy and signal persistence (Piccoli *et al*. 2020). However, traditional experimental methods, such as Surface Plasmon Resonance (SPR) or Bio-Layer Interferometry (BLI), are prohibitively expensive for large-scale screening.

Although recent studies have explored methods to predict antigen-antibody affinity (Li *et al*. 2025) or binding free energy (Hummer *et al*. 2025) to assist in high-throughput screening, end-to-end deep learning methods face several critical challenges. First, the availability of high-quality antigen-antibody complexes paired with experimental affinity or kinetic data is extremely limited compared to the vast space of single sequences. This scarcity makes it difficult to construct high-performing and generalizable expert prediction models using only raw data. Second, beyond static affinity ($K_{d}$), binding kinetics $k_{\mathrm{off}}$ are determinants of drug efficacy and safety (e.g. residence time) (Lee *et al*. 2019). Predicting $k_{\mathrm{off}}$ is notoriously difficult due to the even greater scarcity of data and the complex temporal dynamics of molecular unbinding. Existing antibody screening tools often neglect these biological behaviors associated with efficacy persistence, limiting the practicality for real-world virtual screening. To address the challenges, we propose AbAgKer, a novel model for antigen-antibody interaction prediction. We incorporate pre-trained representations and design multiple sub-modules guided by biological prior knowledge to reduce interaction modeling difficulty. To tackle the scarcity of activity data, we employ semi-supervised learning, significantly expanding the training dataset and achieving stable affinity prediction. Experimental evaluations indicate that our model achieves the best overall performance among the evaluated baselines. Finally, to address the lack of computational screening methods for kinetic metrics in antibody engineering, we propose leveraging the learned binding interaction representations to predict the dissociation rate constant $k_{\mathrm{off}}$. In summary, we provide an effective screening method for predicting the strength of antigen-antibody interactions and their functional persistence.

## 2 Methodology

### 2.1 Overview

The architecture of AbAgKer is illustrated in Fig. 1a. First, we extract residue-level representations for both the antigen and antibody by embedding their paired sequences using domain-specific PLMs. Next, the antigen residue-level representations are fed into the Multi-scale feature adaptive Epitope Identification (MEI) module to delineate binding epitope regions within complex antigen proteins. Subsequently, we design the CDR-Guided Global-Local Attention (CAttention) mechanism to perform antibody feature re-fusion and extract CDR loop representations from antibody chains. Finally, the pooled representations of the antigen and antibody are concatenated and input into a Mixture-of-Experts (MoE) architecture (Rajbhandari *et al*. 2022). The MoE architecture facilitates fine-grained representation learning for diverse antigen-antibody binding scenarios. The results from multiple experts are then input into an MLP regression head to predict interaction affinity and kinetic information.

### 2.2 Pre-trained protein language model representation

To capture the rich semantic information hidden in protein sequences, we employ state-of-the-art PLMs as the feature backbone. For the Antibody Encoder, we use PALM-H3 (He *et al*. 2024) specifically optimized for antibodies. Specifically, we employ its Heavy-chain RoFormer and Light-chain RoFormer to encode heavy and light-chain sequences, respectively. Unlike general protein models, PALM-H3 is pre-trained on massive antibody repertoires, effectively capturing recombination patterns and loop hypervariability. For the antigen encoder, we employ ESM-2 (Lin *et al*. 2023), a transformer protein language model trained on millions of diverse protein sequences. We use the residue-level representation from the pre-trained protein language models. To handle variable-length sequences of antibodies and antigens, we use the truncation and padding strategies from the protein tokenizer to maintain fixed-length inputs for the neural network. The heavy- and light-chain residue embeddings from PALM-H3 are concatenated along the sequence dimension to form the antibody representation $X_{Ab}\in R^{L_{Ab}\times d_{Ab}}$, where $L_{Ab}$ and $d_{Ab}$ are set to 512 and 768. The antigen encoder provides evolutionary-rich residue representations $X_{Ag}\in R^{L_{Ag}\times d_{Ag}}$ for characterizing antigen epitope features, where $L_{Ag}$ and $d_{Ag}$ are set to 896 and 640, respectively. Then, these residue-level representations are projected into a unified hidden dimension *d* of 512 via a linear layer before being passed to the downstream modeling modules.

### 2.3 Multi-scale feature adaptive epitope identification module

Antigen epitopes are often composed of discontinuous residues that form specific 3D structural motifs, and the standard global pooling operations tend to obscure this fine-grained structural information. To address this, we propose the MEI module, which dynamically fuses the antigen’s PLM embedding with pre-calculated sequence pseudo-structure features to identify the latent binding residue location of the antigen. As shown in Fig. 1b, we use the encoder to obtain residue-level embeddings for the antigen, projecting the features to $X_{Ag}\in R^{L_{Ag}\times d}$, where *d* is the token representation dimension of 512. Second, the sequence-based physicochemical property feature shows a great capability for epitope prediction (Wang *et al*. 2026). Therefore, we incorporate biological prior knowledge to pre-analyze potential binding information at the sequence level for locating possibly binding token embeddings of conformational or linear epitopes. We employ the bioinformatics toolkit B2bTools (Kagami *et al*. 2021) to calculate six physicochemical and structural features from the antigen sequence, including the backbone disordered properties (Orlando *et al*. 2022), sidechain properties (Cilia *et al*. 2013), and early folding propensity (Raimondi *et al*. 2017). Additionally, we design a mask vector within the maximum sequence length to mark the antigen’s valid length. These are stacked to construct the sequence pseudo-structure feature matrix $S_{\mathrm{sef}}$. Subsequently, we employ a fused gated weighting mechanism via a dual-path architecture that combines local residue context and biological sequence bias.

Firstly, we apply a convolution operation with a kernel size of 9 in the token dimension of the antigen representation to capture local residue weight $w_{\mathrm{conv}}$ from antigen fragment context:


$$w_{\mathrm{conv}}=\text{Conv}(X_{Ag})\in R^{L_{Ag}\times 1}$$

Then, we design a learnable projection to extract pseudo-structural information for the per-residue property weight $w_{\mathrm{sef}}$ of antigen from the pre-calculated matrix $S_{\mathrm{sef}}$ at the residue level:


$$w_{\mathrm{sef}}=S_{\mathrm{sef}}\cdot W_{s}+b_{s}$$

where $W_{s}\in R^{7\times 1}$ is a learnable parameter matrix, $b_{s}$ is the bias term.

The final epitope token weights $w_{\mathrm{epitope}}$ are then adaptively fused via a learnable gate, defined as:


$$w_{\mathrm{epitope}}=\text{Softmax}\left(\text{Tanh}\left(\alpha \cdot w_{\mathrm{conv}}+(1-\alpha )\cdot w_{\mathrm{sef}}\right)\right)$$

where α represents the learnable weight, and Tanh is the activation function.

Finally, we perform Top-K pooling based on importance weight $w_{\mathrm{epitope}}$ to select the most significant *K* tokens, yielding $Ag_{\mathrm{pool}}\in R^{K\times d}$, where *K* is 64.

### 2.4 CDR-guided global-local attention mechanism

**Figure 2:**
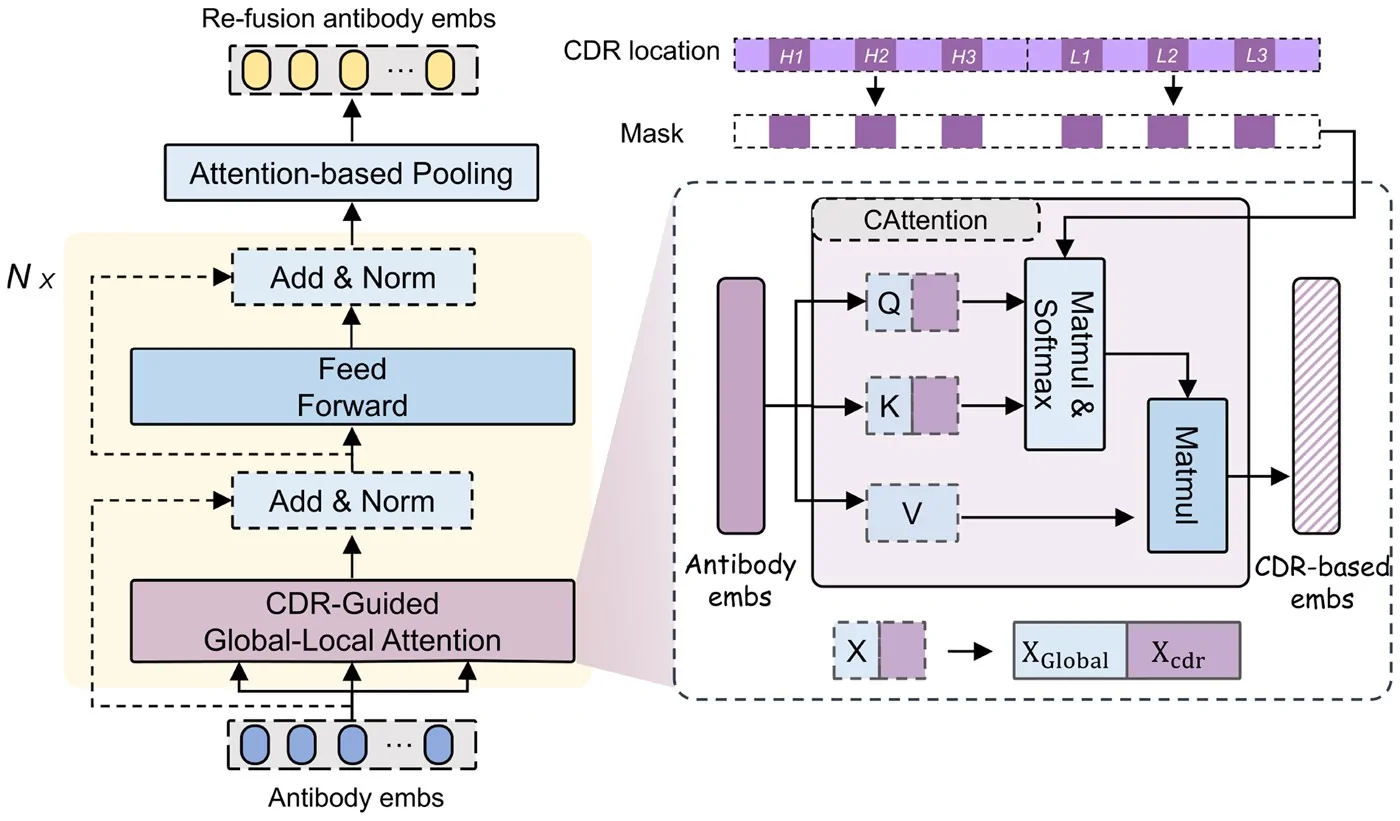
The CAttention encoder module and CAttention mechanism, where the $X_{\mathrm{cdr}}$ is the CDR local attention heads, and $X_{\mathrm{Global}}$ is the global attention heads. The CAttention encoder is composed of multiple layers of CAttention, and its output is the re-encoded antibody representation.

Antigen-antibody binding is predominantly determined by the complementarity-determining regions (Pantazes and Maranas 2010). To leverage this, as shown in Fig. 2, we propose a novel CAttention mechanism that reinforces the model’s focus on antibody CDR token representations. Unlike standard attention mechanisms (Vaswani *et al*. 2017), we explicitly define a mask tensor *M* from antibody CDR information to constrain attention in specific heads. We set *M* to negative infinity when the position is not the CDR residue, and to 0 otherwise. We construct a multi-layer encoder architecture based on CAttention to learn CDR-guided re-encoding of the antibody representation. The CAttention is constrained to enhance focus on antibody CDR regions via CDR-local heads, effectively forcing the model to prioritize paratope features. The CAttention head outputs are calculated as:


$$\text{CAttention}(Q_{i},K_{i},V_{i})=\text{Softmax}\left(\frac{Q_{i}K_{i}^{T}}{\sqrt{d_{k}}}+B_{i}\right)V_{i}$$

where $Q_{i},K_{i},V_{i}$ are derived from $X_{Ab}$ via different learnable weights, $d_{k}$ is the dimension of each attention head, and $B_{i}$ is the bias term for the *i*-th attention head, if i<n/2, the $B_{i}$ is set to a zero tensor (using global attention mechanisms), the remaining heads set $B_{i}$ as *M* (using the CDR local attention mechanisms). The *n* denotes the total number of heads.

Then, we design a 4-layer CAttention-based encoder to rebuild the new antibody representation $X_{Ab}$, which is the architecture shown in Fig. 2. Finally, we apply Top-K pooling to the output attention scores $w_{\mathrm{score}}$ of the last CAttention layer and antibody representation $X_{Ab}$ to extract the top *K* token embedding with the highest weights:


$$Ab_{\mathrm{pool}}=\text{Top}-K(X_{Ab},w_{\mathrm{score}})$$

where $Ab_{\mathrm{pool}}\in R^{K\times d}$ serves as the final refined antibody representation tensor.

### 2.5 Bi-directional interaction and MoE-based prediction

To capture the induced fit mechanism (Fernández-Quintero *et al*. 2020) governing antigen-antibody binding, we introduce a bi-directional cross-attention module. This module consists of two symmetric cross-attention units: one in which the antibody representation $Ab_{\mathrm{pool}}$ serves as the query to attend to the antigen $Ag_{\mathrm{pool}}$, and vice versa. The two directional outputs are concatenated and then mean-pooled over all tokens to generate the fused representation $x\in R^{d}$. Given the vast diversity of antigen types (e.g. viral proteins, membrane proteins), relying on a single interaction mode makes it difficult to capture the complex biological binding patterns. To address this, we introduce an MoE architecture, comprising expert networks and a gating network. The gating network computes a probability distribution over the experts to dynamically route inputs based on the antigen-antibody interaction representation $H_{\mathrm{interaction}}$, which is computed as a weighted sum of the expert outputs:


$$H_{\mathrm{interaction}}=\sum_{i=1}^{N}G{\left(x\right)}_{i}\cdot E_{i}(x)$$

where the *N* of 4 denotes the number of experts, $E_{i}$ represents the *i*-th expert network (implemented as a two-layer feed-forward network), and $G{\left(x\right)}_{i}$ is the gating weight assigned to the *i*-th expert. This mechanism enables the dynamic selection of specific experts to infer the specific binding mode and interaction strength for the current antigen-antibody pair. Finally, we employ an MLP as the regression head to predict the affinity values.

## 3 Experimental results

### 3.1 Experimental setup

#### 3.1.1 Datasets

Our primary antigen-antibody datasets stem from the SKEMPI v2.0 (Jankauskaitė *et al*. 2019) and SAbDab (Dunbar *et al*. 2014) public databases. SKEMPI v2.0 contains wild-type and mutant protein-protein interaction complex data and corresponding wet-lab results (e.g. $K_{d}$, $k_{\mathrm{off}}$), and includes a subset of antigen-antibody samples. Different from the traditional use of SKEMPI v2.0 database, we extracted the wild-type or mutant antigen-antibody complexes as a separate sample when their corresponding experimental values were available. The affinity label of each sample is the experimentally measured $K_{d}$ value of that specific wild-type or mutant complex. SAbDab is a comprehensive database dedicated to antibodies, recording the structures of antigen-antibody complexes and corresponding binding affinity data. Due to B2bTools’ suite requiring a minimum of 5 residues for antigen sequence feature extraction (specifically for components like DynaMine), and to further reduce noise from very short fragments, we adopted a stricter minimum antigen length of 20 residues. Meanwhile, to accommodate PLMs input requirements, we applied length constraints: antigens between 20 and 896 residues, and each of the heavy and light chains with a maximum sequence length of 256 residues. We collected all the antigen-antibody datasets from SKEMPI v2.0 and SAbDab, and curated three specific sub-datasets based on the selected antigen-antibody data: AbAgA, AbAgI, and AbAgKoff. First, we created the AbAgA dataset, which comprises samples with labeled affinity ($K_{d}$) for modeling the affinity prediction task. Second, the AbAgI is a large dataset of antigen-antibody pairs without experimental results, which was used for the semi-supervised pseudo-labeling phase. Third, we curated a specialized AbAgKoff dataset by integrating experimental kinetic data for the $k_{\mathrm{off}}$ prediction task. We employed CD-HIT (Li and Godzik 2006) to compute the dataset statistics summarized in Table 1. Notably, antigen clustering was performed only to describe antigen sequence redundancy, and the actual dataset split was strictly based on antibody heavy-chain sequence clusters at 95% identity. In the Antigen and Antibody columns, we also report the original number of unique valid sequences, alongside the final number of CD-HIT clusters for the antigen or antibody heavy chain in parentheses. All experimental values were standardized to the negative logarithmic scale (e.g. $pK_{d}$, $pk_{\mathrm{off}}$) for model training.

**Table 1:** Dataset statistics and clustering results.

Dataset	Antigen	Antibody	Available Pairs
SAbDab	6899(2023)	6930(3949)	9524
SKEMPI v2.0	327(28)	421(41)	1217
AbAgA	976(351)	1058(452)	1953
AbAgI	5596(1705)	5485(3260)	7725
AbAgKoff	119(16)	85(18)	272

For the Antigen and Antibody columns, values are reported as ‘number of unique sequences (number of CD-HIT clusters)’. The Available Pairs column indicates the number of valid antigen-antibody sample pairs retained after data preprocessing.

#### 3.1.2 Baseline models and evaluation metrics

In this study, we compared five deep learning affinity prediction baseline models across our datasets. Following the prior study (He *et al*. 2024), we compare with A2binder, ESM-F, and AntiBERTa2. The A2binder (He *et al*. 2024) combines PALM-H3 antibody embeddings with ESM-2 antigen features via a Multi-Fusion Convolutional Neural Network (MF-CNN) for affinity prediction. We also compare with DG-Affinity (Yuan *et al*. 2023), which fuses TAPE antigen features and AbLang antibody features using a ConvNeXt backbone. To assess the capability of diverse antibody embedding, we also implement an AntiBERTy-F baseline model that replaces the antibody representation encoder with AntiBERTy (Ruffolo *et al*. 2021) within the A2binder architecture. We use MSE (Mean Squared Error), RMSE (Root Mean Squared Error), and Pearson (Pearson Correlation Coefficient). Besides, to evaluate the ranking capability for affinity differences, we also used Spearman (Spearman rank correlation coefficient) and CI (Concordance Index) for comprehensive comparison.

#### 3.1.3 Training protocol and hyperparameters

We evaluated model performance using five-fold cross-validation. For each fold, the model was trained on four folds and evaluated on the remaining held-out validation fold. This procedure was repeated five times, with each fold used once as the validation fold. During training, the checkpoint with the lowest RMSE on the held-out validation fold was saved, and the final performance was reported as the average and standard deviation of the validation-fold results across the five folds. To ensure comparability of study results, the same dataset splitting method was used for all baselines. In the semi-supervised learning experiment, pseudo-labeled AbAgI samples were added only to the corresponding AbAgA training folds, while the held-out validation fold was kept unchanged for evaluation.

To prevent mode collapse in the MoE module that selects only one expert, we introduced an auxiliary load-balancing loss based on the variance of the importance scores (Shazeer *et al*. 2017). Finally, we adopted a combined loss comprising the MSE loss and the MoE auxiliary loss for model training. Our model was optimized using the Adam optimizer. The maximum number of epochs was set to 50. For all experiments, we employed a peak learning rate of $5\times 10^{-5}$ and a minimum learning rate of $1\times 10^{-6}$. For the affinity and kinetics prediction tasks, the batch size was set to 8; when incorporating semi-supervised learning to enhance performance, it was increased to 16. Our learning rate schedule consisted of a linear warm-up phase spanning the first 10% of the total training steps, followed by a cosine annealing decay.

### 3.2 Comparison of model performance with baselines

We evaluated AbAgKer against baseline models on the AbAgA dataset, and the results are presented in Table 2. All results are obtained under the same five-fold cross-validation protocol. Overall, AbAgKer achieves the best average performance across all five evaluation metrics, demonstrating its ability to predict antigen-antibody binding affinity for diverse complexes. Specifically, we benchmarked the proposed AbAgKer against five representative baseline models, encompassing general protein language models (ESM-F) and antibody-specific pre-trained models (A2binder, AntiBERTa2, AntiBERTy-F, DG-Affinity). Notably, our model achieves Pearson and Spearman correlations of 0.4483 and 0.4168, respectively, surpassing the second-best AntiBERTy-F. Furthermore, AbAgKer attains the lowest error rates, with an RMSE of 1.3774 and an MSE of 1.9067, indicating that our predicted $K_{d}$ values align most closely with experimental ground truth. Moreover, our model also achieves 0.6463 in CI, further validating its robustness in correctly distinguishing binding affinity ranks across random pairs. While antibody-specific models like AntiBERTa2 and AntiBERTy-F achieve competitive performance through training on massive antibody repertoires, AbAgKer still outperforms them. We attribute this improvement to our bi-directional interaction design. Unlike baselines that primarily encode antibody sequences implicitly or independently, AbAgKer explicitly models the interaction space between the antibody paratope and the antigen epitope via the bi-cross-attention block. Compared to ESM-F, AbAgKer shows consistent improvements. It validates the effectiveness of our MEI Module and CAttention, which successfully inject biological inductive biases. The CAttention module allows the model to focus on the most relevant binding regions rather than the entire sequence. As shown in Fig. S1, available as supplementary data at *Bioinformatics* online, we also visualized the performance of AbAgKer alongside the baseline models using scatter plots of predicted versus experimental values. Compared with the baseline models, AbAgKer shows better overall agreement between predicted and experimental affinity values, as reflected by its higher CI and Pearson correlation and lower RMSE, also demonstrating the effectiveness of our framework.

**Table 2:** Performance comparison on the antigen-antibody affinity prediction benchmark.

Model	**MSE(** ↓ **)**	**RMSE(** ↓ **)**	**Pearson(** ↑ **)**	**Spearman(** ↑ **)**	**CI(** ↑ **)**
A2binder	2.0523 (0.1812)	1.4315 (0.0632)	0.3796 (0.1210)	0.3537 (0.1168)	0.6235 (0.0438)
AntiBERTa2	2.0509 (0.1576)	1.4312 (0.0553)	0.3629 (0.1414)	0.3173 (0.1604)	0.6102 (0.0562)
DG-Affinity	2.0041 (0.1368)	1.4150 (0.0485)	0.3404 (0.1830)	0.3284 (0.1457)	0.6171 (0.0512)
ESM-F	2.1312 (0.2752)	1.4575 (0.0931)	0.3717 (0.1232)	0.3450 (0.1317)	0.6198 (0.0480)
AntiBERTy-F	1.9728 (0.1498)	1.4038 (0.0530)	0.4066 (0.1071)	0.3742 (0.1026)	0.6302 (0.0400)
AbAgKer	**1.9067 (0.3039)**	**1.3774 (0.1079)**	**0.4483 (0.1551)**	**0.4168 (0.1718)**	**0.6463 (0.0653)**
AbAgKer_wAbAgI_	1.2865 (0.2240)	1.1309 (0.0966)	0.6754 (0.0928)	0.6541 (0.1115)	0.7409 (0.0439)
AbAgKer_Koff_	0.7166 (0.4981)	0.8147 (0.2572)	0.5425 (0.1931)	0.5354 (0.3407)	0.7013 (0.1319)

The best and second-best results are highlighted in bold and underlined, respectively. Values denote mean (standard deviation) across five folds. The results for AbAgKer_wAbAgI_ and AbAgKer_Koff_ models are also presented. Note that AbAgKer_Koff_ is evaluated on the AbAgKoff dataset for *pk*_*off*_ prediction and is therefore not directly comparable to the affinity results of the other models.

### 3.3 Ablation study

**Figure 3:**
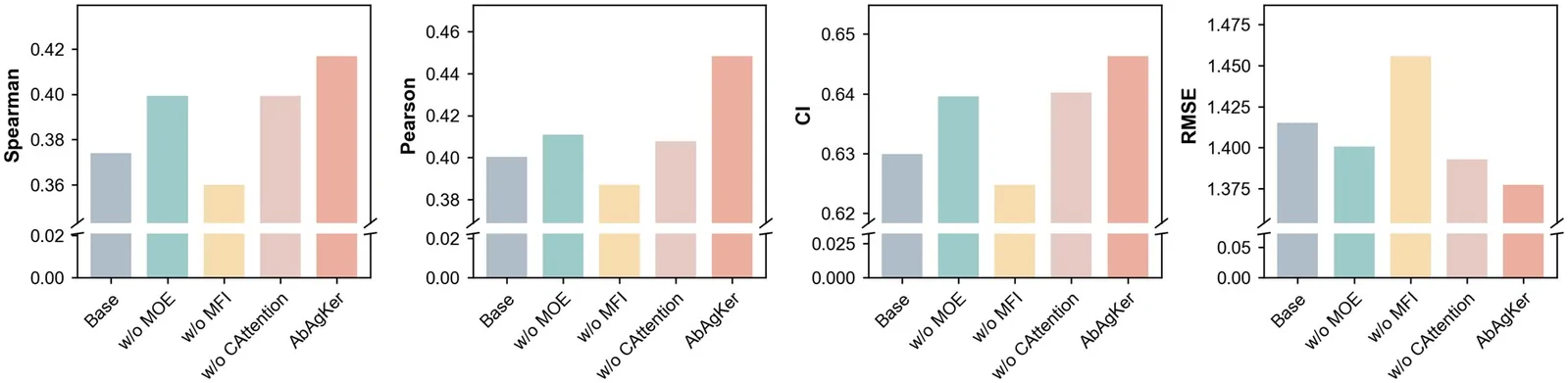
Performance comparisons of AbAgKer with its variants on the held-out validation folds under five-fold cross-validation. The bar chart is grouped by evaluation metric, and colors indicate different model variants. Lower values are better for RMSE, whereas higher values are better for Pearson, Spearman, and CI.

To validate the contributions of the CAttention, MEI Module, and MoE module, we conducted a comprehensive ablation study on the AbAgA dataset. We compared the AbAgKer framework against a “Base” model (comprising the PLM backbones and the Linear layer) and three variants in which specific modules were removed (“w/o”). The results are summarized in Fig. 3. As illustrated, AbAgKer consistently outperforms all ablation variants across the evaluation metrics, achieving the highest Pearson and the lowest RMSE, by 0.4483 and 1.3774, respectively. The substantial gap between the Base and the AbAgKer model indicates a strong synergistic effect with a 10.2% relative improvement in Spearman correlation, and underlines that simply using and concatenating pre-trained language models is insufficient. The structural inductive biases introduced by our CAttention and MEI modules, combined with the dynamic routing of the MoE, are important for capturing the fine-grained physicochemical patterns governing binding affinity. Analyzing specific components, we observe that removing the CAttention module results in a performance drop, with the Pearson correlation declining by approximately 9.07%. It highlights the usefulness of explicitly attending to the antibody complementarity-determining regions. Without this mechanism, the model treats the sequence uniformly, allowing non-binding framework regions to introduce noise that compromises the antibody representation. Similarly, removing the MEI and MoE modules results in Spearman correlations of 0.3870 and 0.4073, respectively. The drop in the ‘w/o MEI’ variant demonstrates that the MEI module effectively delineates the valid binding interface by integrating sequence pseudo-structure feature information, preventing the dilution of epitope and the vast non-binding regions features. Furthermore, the decline in the ‘w/o MoE’ variant demonstrates that a single MLP head is inadequate to model the complex and multimodal nature of antigen-antibody binding landscapes, whereas the MoE architecture successfully captures these diverse interaction modes.

### 3.4 Performance boost via semi-supervised self-distillation strategy

The scarcity of experimentally labeled affinity data remains a fundamental bottleneck in deep learning-based affinity prediction. To address this, we introduce semi-supervised learning by fusing the unlabeled antigen-antibody binding dataset AbAgI. For the pseudo-label generation of self-distillation, we trained a base model on the AbAgA training folds and used it to predict affinity values for the unlabeled AbAgI samples. Then, for each cross-validation fold, pseudo-labeled AbAgI samples were added only to the corresponding training folds to train the AbAgKer${\;}_{\text{wAbAgI}}$ model, and the held-out validation fold from AbAgA was kept unchanged and used for evaluation. Specifically, the model was trained from scratch, and equal weights were assigned to both the experimental and pseudo-labeled samples.

**Figure 4:**
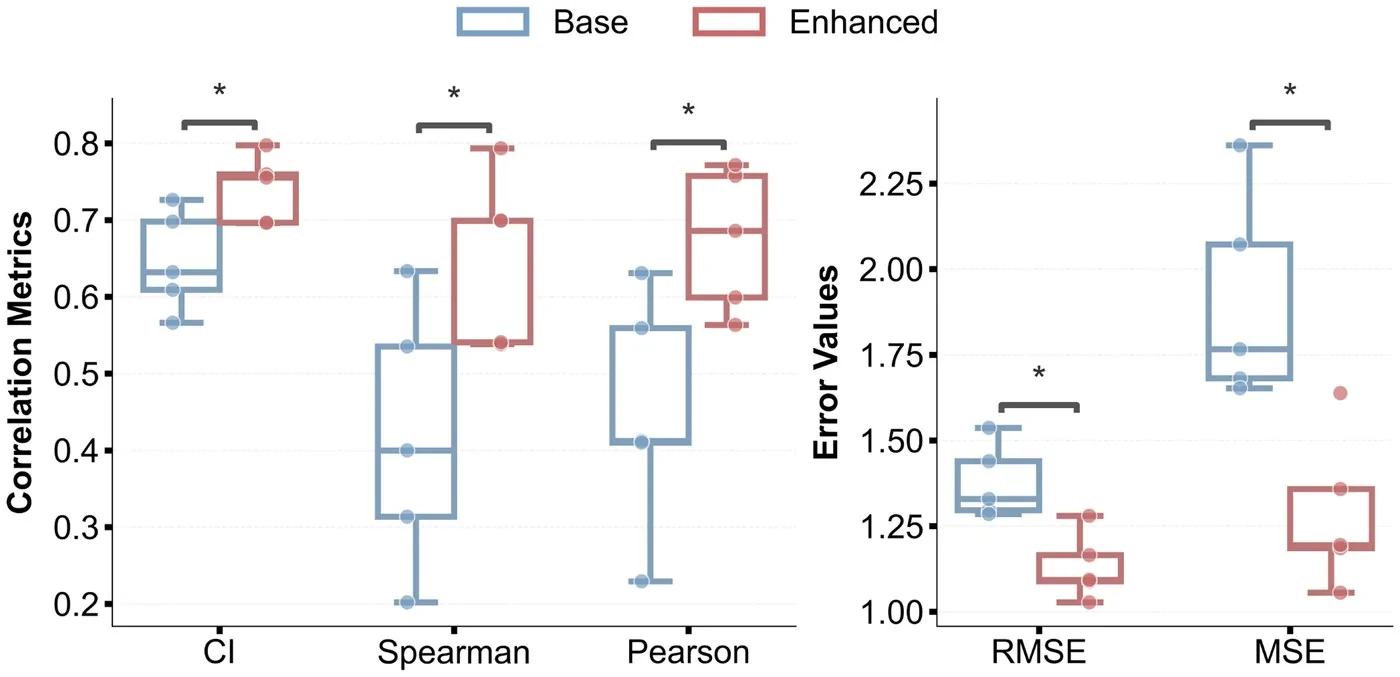
Visualization results of box plots for AbAgKer (Base) and AbAgKer${\;}_{\text{wAbAgI}}$ (Enhanced) Model.

**Figure 5:**
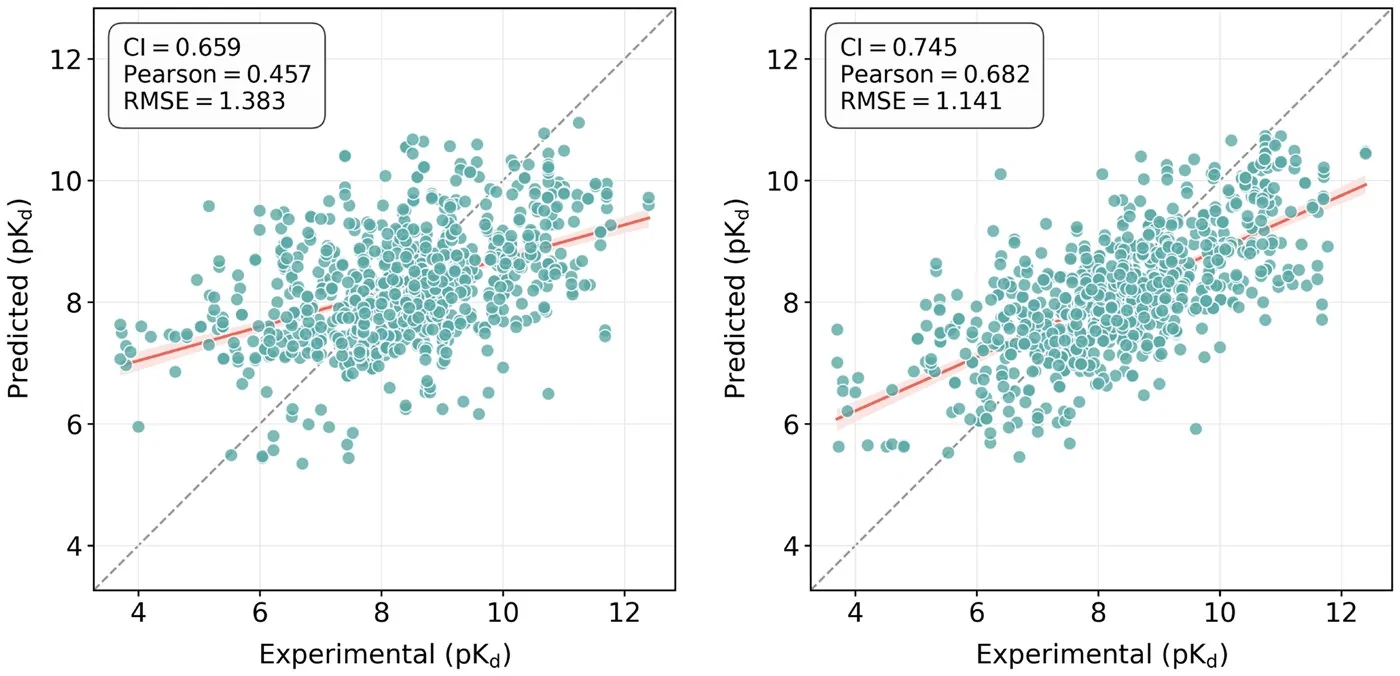
Scatter plots of experimental versus predicted affinity values for AbAgKer (left) and AbAgKer${\;}_{\text{wAbAgI}}$ (right). Points are pooled from all held-out validation folds under five-fold cross-validation.

We demonstrate that the semi-supervised learning strategy achieved substantial improvement. As shown in Fig. 4, we visualize all AbAgKer${\;}_{\text{wAbAgI}}$ and AbAgKer predictions across all held-out validation folds in the five-fold cross-validation setting. The results reveal a substantial improvement in predictive capability by the AbAgKer${\;}_{\text{wAbAgI}}$ model. Compared to the base AbAgKer trained solely on labeled data, AbAgKer${\;}_{\text{wAbAgI}}$ achieves a dramatic performance boost across all metrics. Specifically, the Spearman and Pearson results of AbAgKer${\;}_{\text{wAbAgI}}$ improved to 0.6541 and 0.6754. Concurrently, the prediction error is significantly reduced, with RMSE dropping from 1.3774 to 1.1309. AbAgKer${\;}_{\text{wAbAgI}}$ surpasses the best baseline model AntiBERTy-F by 0.2799 in Spearman and reduces its MSE by approximately 35%. The improvement by the base model captures the fundamental physics of binding via its architectural inductive biases (MEI and CAttention module). However, it is constrained by the limited size of the labeled training set. Then, incorporating these pseudo-labeled samples into the training set allows the model to encounter a significantly more diverse array of antigen representations and complex binding pairs, thereby learning more robust and generalizable representations of binding interaction landscapes. As shown in Fig. 5, we also compare the prediction results of AbAgKer${\;}_{\text{wAbAgI}}$ and the AbAgKer model by a scatter plot. Compared to the base model, these plots show a significantly tighter clustering of data points around the identity line. This visual tightening corresponds to a substantial improvement in Spearman and MSE. The reduction in outliers in these plots highlights the effectiveness of incorporating pseudo-labeled samples from the AbAgI dataset. These results also demonstrate the effectiveness of the semi-supervised self-distillation strategy for antigen-antibody affinity data scarcity.

### 3.5 Few-shot learning for antigen-antibody kinetics prediction

The dissociation rate constant is a critical pharmacokinetic parameter that determines the drug residence time on the target (Lee *et al*. 2019). Antibodies with slower off-rates often exhibit superior therapeutic efficacy and an extended duration of action. However, measuring kinetic constants is time-consuming and expensive, leading to severe data scarcity that hinders the development of data-driven predictors. To address this, we propose a few-shot transfer learning paradigm. We hypothesize that the AbAgKer encoder, pre-trained on the affinity task, has successfully captured the fundamental physicochemical principles governing antigen-antibody interactions. Consequently, the learned representations should be transferable to downstream kinetic tasks. Methodologically, we freeze the parameters of the trained AbAgKer${\;}_{\text{wAbAgI}}$ to serve as a static feature extractor and append an MLP regression head as the prediction head. This AbAgKer${\;}_{\text{Koff}}$ model is trained on the AbAgKoff dataset (As shown in Fig. 1d). As this represents a pioneering effort in deep learning-based antigen-antibody kinetics prediction, there are no established baselines for direct comparison. We only report the performance of AbAgKer${\;}_{\text{Koff}}$ to establish a high-quality benchmark for the community.

As shown in Table 2, the model achieves promising performance despite the limited data. The AbAgKer${\;}_{\text{Koff}}$ model attains average Pearson and Spearman correlations of 0.5425 and 0.5354, respectively. Given the very limited size of the AbAgKoff dataset, we observe a relatively large variance across folds; nevertheless, the consistently positive correlations suggest that the interaction representations learned by AbAgKer carry transferable kinetic signal. Furthermore, since the equilibrium dissociation constant is defined as $K_{d}=k_{\mathrm{off}}/k_{on}$, jointly predicting $K_{d}$ and $k_{\mathrm{off}}$ could in principle allow the association rate constant ($k_{on}$) to be derived, pointing to a potential direction for comprehensive kinetic analysis. These results show that our model is not only an affinity predictor but also a comprehensive kinetic analysis tool, capable of supporting the full spectrum of screening and optimization in computational antibody discovery.

## 4 Discussion

This study presents a semi-supervised learning framework for antigen-antibody affinity and kinetics prediction. Our results show that pre-trained representations and biological prior guidance can support the modeling of antigen-antibody affinity and kinetics, and suggest that leveraging unlabeled antigen-antibody interaction pairs through self-distillation is a promising strategy for addressing the data scarcity of affinity-labeled data. Furthermore, we explored transferring the learned interaction representations to kinetics prediction via few-shot learning.

Nevertheless, as detailed in Supplementary Section B (Figs. S2 and S3, available as supplementary data at *Bioinformatics* online), the current framework also faces additional challenges when extended to fine-grained prediction of mutation-induced affinity changes within highly homologous antibody sequence space. Although PLM-derived token-level representations are effective at capturing global sequence semantics, they may be less sensitive to the subtle local physicochemical perturbations caused by amino acid mutations. While PLMs have substantially reduced the reliance on large labeled datasets in many downstream tasks, their representational limitations may still constrain performance on fine-grained antibody tasks. In the future, the development of more advanced PLM pre-training paradigms and fine-grained affinity modeling architectures will remain important for antigen-antibody interaction analysis. In particular, future affinity prediction work may benefit from explicitly modeling wild-type and mutant pairs and from introducing mutation-aware or delta-based affinity objectives to better capture subtle local perturbations. With the continued improvements in PLM representation capability and dataset scale, more accurate antigen-antibody predictive models may further accelerate antibody discovery and optimization.

## 5 Conclusion

In this paper, we propose a comprehensive framework, AbAgKer, for antigen-antibody interaction prediction. By integrating domain-optimized PLMs with the novel MEI module and CDR-guided attention, our model effectively learns the intricate mapping between sequence information and binding affinity. The proposed semi-supervised pseudo-labeling strategy and the MoE-based prediction module further enhance the model’s generalization capability and robustness. Notably, we extended the utility of AbAgKer to predict dissociation rates and achieved promising results with minimal data. Experiments across multiple metrics demonstrate that AbAgKer achieves competitive performance among evaluated baselines. Our method provides a robust, high-throughput tool for the in silico screening of antibody candidates.

## Supplementary Material

btag606_Supplementary_Data

## Data Availability

The data underlying this article are publicly available with their sources described in the article.
