# Supplementary material for: AbAgKer: a unified semi-supervised framework for antigen-antibody binding affinity and kinetics prediction
Source: Bioinformatics. 2026 Aug 12;42(8):btag606. doi: 10.1093/bioinformatics/btag606 (PMC13516917; doi:10.1093/bioinformatics/btag606)
Supplement: Supplementary file 1 [file btag606_supplementary_data.pdf]

Supporting Information:

AbAgKer: A Unified Semi-Supervised Framework for Antigen-Antibody Binding Affinity and Kinetics Prediction

Gang Luo[1], Junkai Wang[1], Sizhe Zhang[1], Zhilin Zhu[1], Zhangli Lu[1], and Min Li[1,*]

[1]*School of Computer Science and Engineering, Central South University, Changsha 410083, China*

[*]*Corresponding author, Min Li: limin@mail.csu.edu.cn*

Contents

A Visualization of Predicted and Experimental Affinity Values **2**

B Exploratory Analysis of Mutation Effect Prediction **3**

A. Visualization of Predicted and Experimental Affinity Values

To provide a more detailed view of the model's predictive performance beyond the summary statistics presented in the main manuscript, we present scatter plots comparing the experimentally determined affinity labels (ground truth) with those predicted by AbAgKer and the baseline model. Figure S1 (a-f) displays the scatter plots for each model of the 5-fold prediction results in the AbAgA held-out validation folds. The binding affinity is expressed as pK_d values ($-\log_{10} K_d$). The AbAgKer model predictions exhibit a promising correlation with the experimental ground truth across all samples.

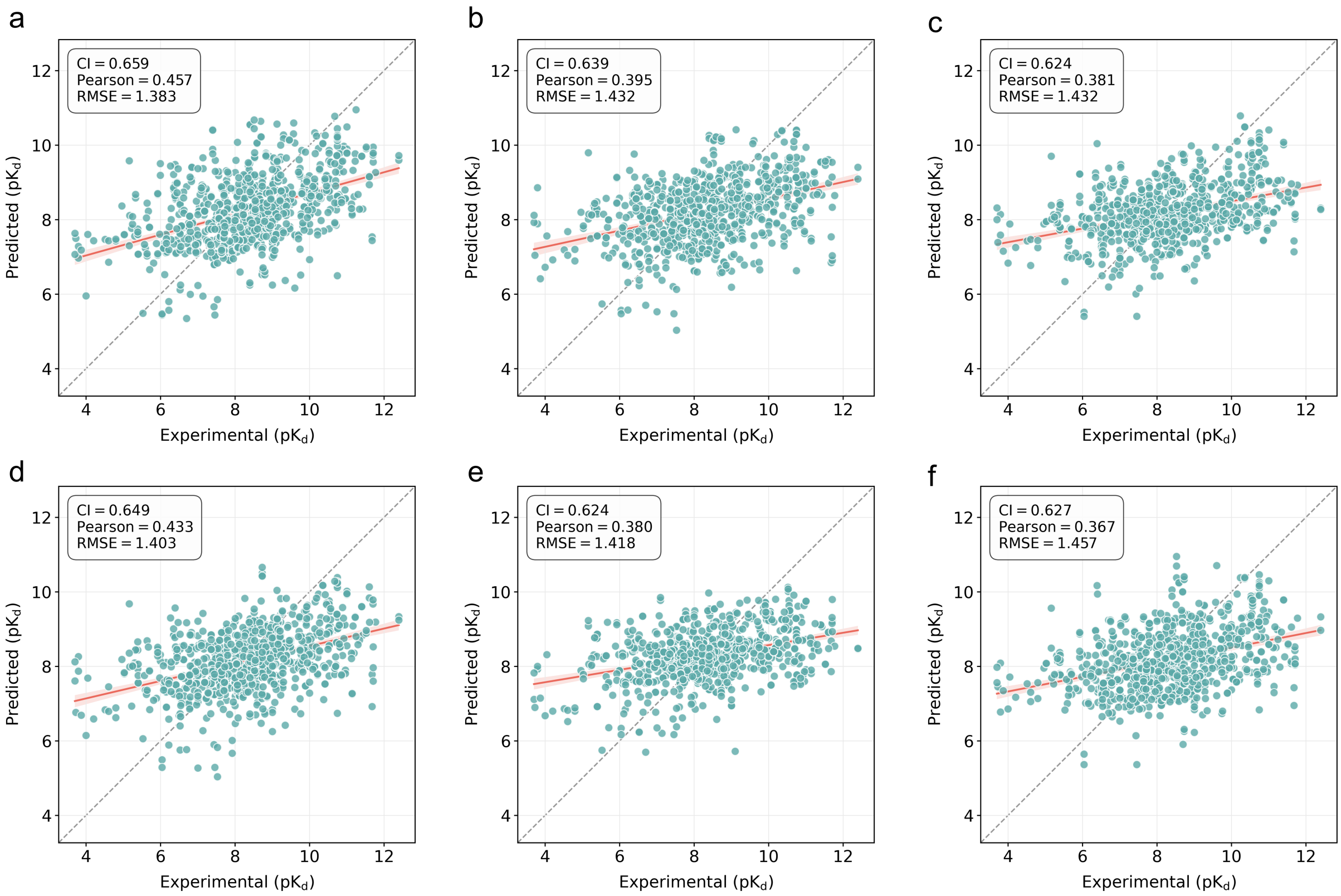

Supplementary Figure S1: Scatter plots of experimental versus predicted affinity values for AbAgKer and baseline models. Points are pooled from all held-out validation folds under five-fold cross-validation. Panels (a-f) correspond to AbAgKer, A2binder, AntiBERTa2, AntiBERTy-F, DG-Affinity, and ESM-F on the AbAgA dataset, respectively.

B. Exploratory Analysis of Mutation Effect Prediction

Antibody optimization hinges on quantifying affinity changes induced by missense point mutations. Here, we apply $\text{AbAgKer}_{\text{wAbAgI}}$ to antibody mutation samples as an exploratory mutation task evaluation for affinity prediction. Unlike general antigen-antibody binding affinity prediction tasks, affinity maturation requires the model to distinguish between beneficial and deleterious mutations within a highly homologous sequence space. We conducted an exploratory analysis of mutation-effect prediction using antibody wild-type and variant samples to assess whether AbAgKer can be useful for predicting mutation affinity. We experimented with the mutation samples selected from the AbAgA held-out validation folds. Notably, we use the CD-HIT tool to implement a rigorous cluster-based split strategy across all datasets to prevent data leakage; there is no overlap between the training and validation sets.

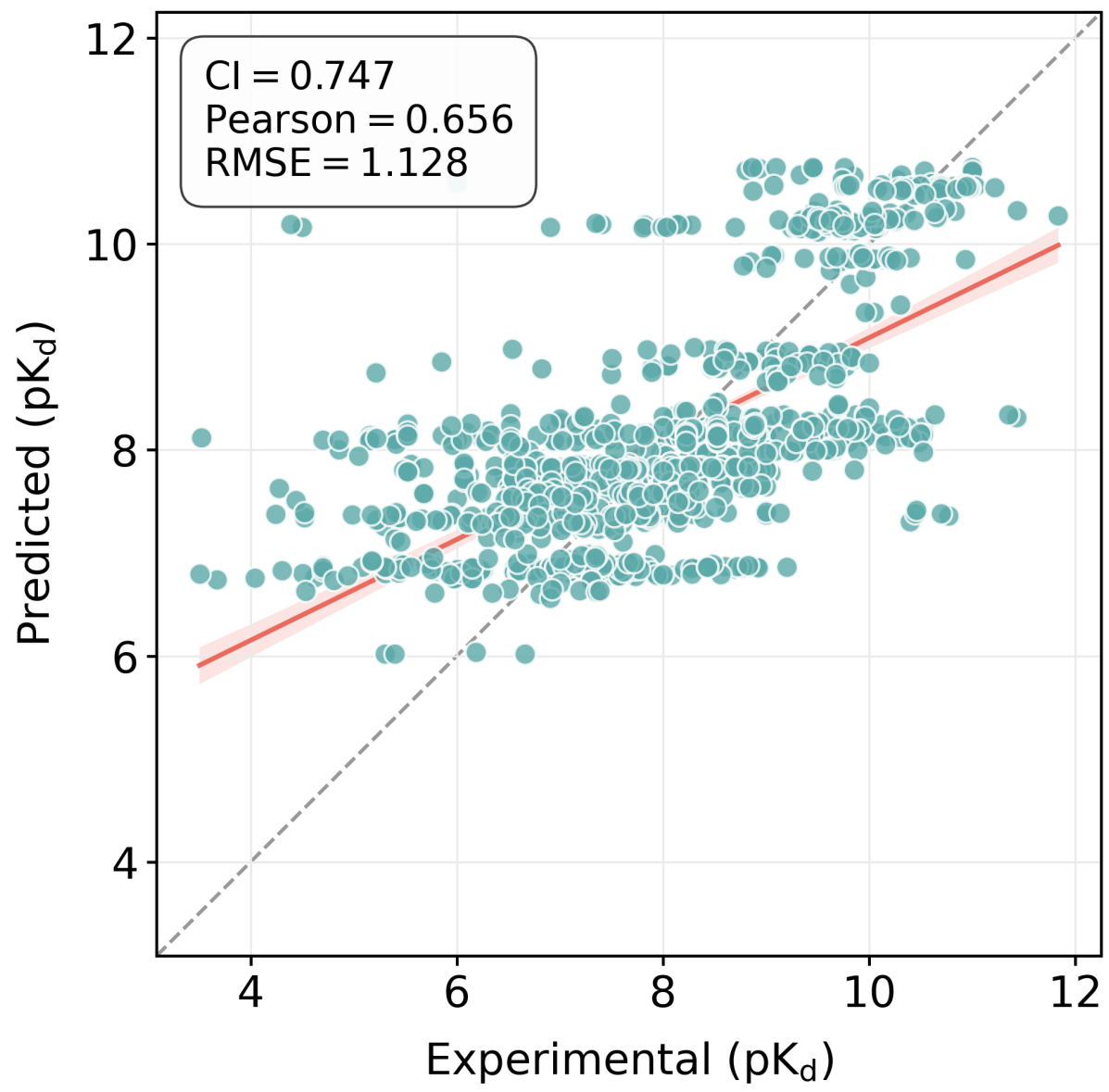

Supplementary Figure S2: Scatter plots of $\text{AbAgKer}_{\text{wAbAgI}}$ on all mutation samples in the AbAgA held-out validation folds.

We evaluate the $\text{AbAgKer}_{\text{wAbAgI}}$ model on the mutation-effect prediction task and present all samples and PDB-based visualization results in Figures S2 and S3, respectively. As shown in Figure S2, the model preserves only limited global trend consistency on the experiment samples, while in the PDB-based results (Figure S3) the predicted values are markedly compressed relative to the experimental distribution. In particular, predictions for variants associated with the same complex tend to collapse into a narrow numerical range, even when the corresponding experimental affinities span a broader interval. This phenomenon indicates that the current model is insufficiently sensitive to small mutation-induced perturbations and is prone to regressing toward a case-level

average prediction. This failure mode is biologically and methodologically informative. Mutation effect prediction requires distinguishing very small local changes against an almost unchanged antigen background and a highly similar antibody scaffold. Under this setting, the dominant signal in the representation may still correspond to the identity of the original complex, whereas the contribution of one or a few residue substitutions can be too weak to substantially alter the final prediction. As a result, the model may correctly recognize that a set of variants belongs to the same parent complex, while still failing to resolve the fine-grained ranking among individual mutants. Meanwhile, the limited size of mutation datasets and the narrow sequence diversity within each mutational series have further exacerbated this problem. This also reflects a limitation of current PLMs on mutation-based tasks. Future work may address this limitation by introducing mutation-aware architectures in antibody-antigen affinity prediction.

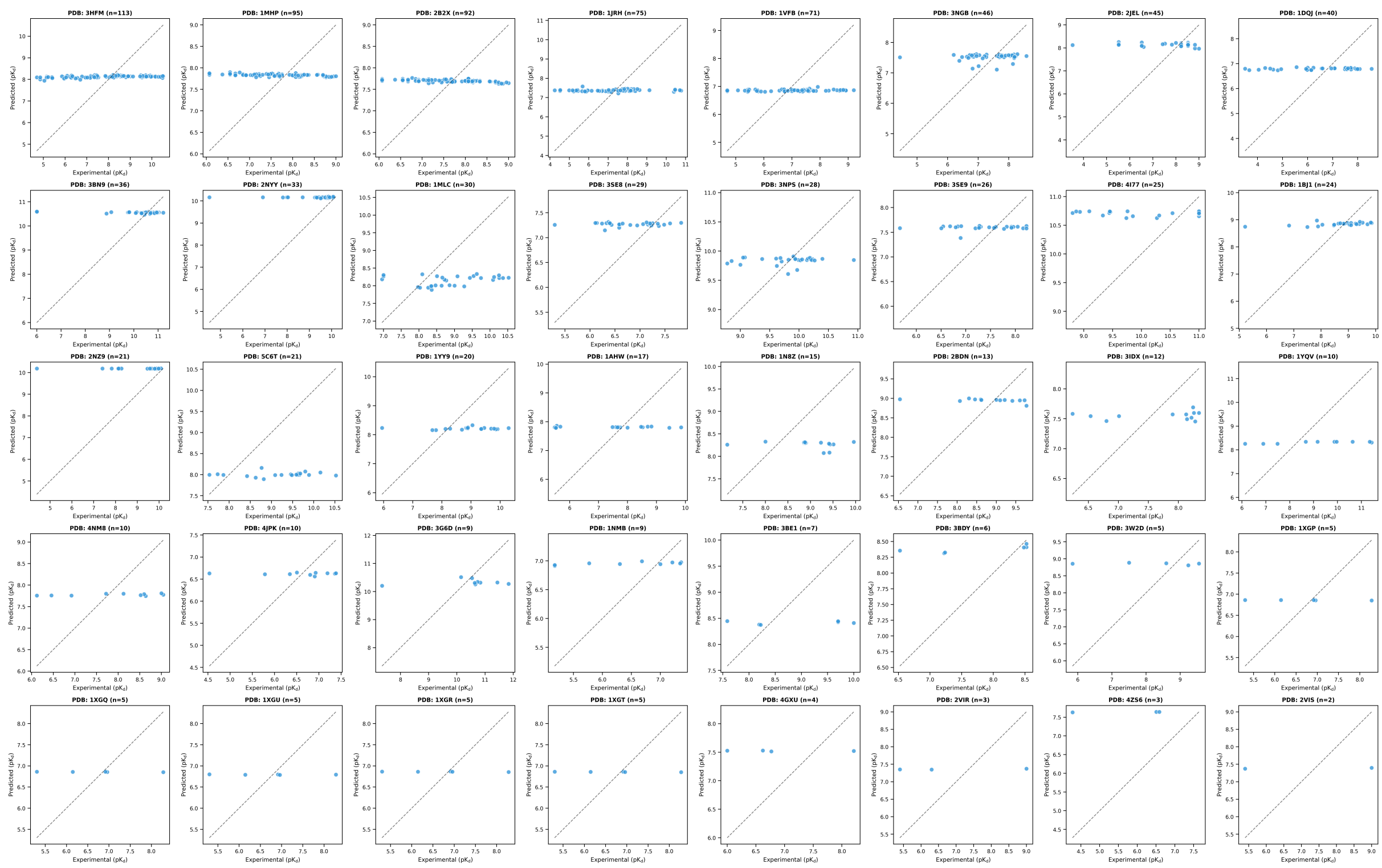

Supplementary Figure S3: Scatter plots of $\text{AbAgKer}_{\text{wAbAgI}}$ on antibody mutation samples in the AbAgA held-out validation folds, grouped by parent complex (PDB entry).
